# In Silico Development of Novel Benzofuran-1,3,4-Oxadiazoles as Lead Inhibitors of *M. tuberculosis* Polyketide Synthase 13

**DOI:** 10.3390/ph16060829

**Published:** 2023-06-01

**Authors:** Ali Irfan, Shah Faisal, Ameer Fawad Zahoor, Razia Noreen, Sami A. Al-Hussain, Burak Tuzun, Rakshanda Javaid, Ahmed A. Elhenawy, Magdi E. A. Zaki, Sajjad Ahmad, Magda H. Abdellattif

**Affiliations:** 1 Department of Chemistry, Government College University Faisalabad, Faisalabad 38000, Pakistan; raialiirfan@gmail.com (A.I.); rakshanda880@gmail.com (R.J.); 2 Department of Chemistry, Islamia College University Peshawar, Peshawar 25120, Pakistan; faisalvbs@gmail.com; 3 Department of Biochemistry, Government College University Faisalabad, Faisalabad 38000, Pakistan; razianoreen@hotmail.com; 4 Department of Chemistry, College of Science, Imam Mohammad Ibn Saud Islamic University (IMSIU), Riyadh 13623, Saudi Arabia; mezaki@imamu.edu.sa; 5 Plant and Animal Production Department, Technical Sciences Vocational School of Sivas, Sivas Cumhuriyet University, Sivas 58140, Turkey; theburaktuzun@yahoo.com; 6 Chemistry Department, Faculty of Science, Al-Azhar University, Nasr City, Cairo 11884, Egypt; elhenawy_sci@hotmail.com; 7 Chemistry Department, Faculty of Science and Art, AlBaha University, Mukhwah, Al Bahah 65731, Saudi Arabia; 8 Department of Health and Biological Sciences, Abasyn University, Peshawar 25000, Pakistan; sajjad.ahmad@abasyn.edu.pk; 9 Department of Chemistry, College of Science, Taif University, Taif 21944, Saudi Arabia; m.hasan@tu.edu.sa

**Keywords:** benzofuran-1,3,4-oxadiazole, tuberclosis, Pks13 inhibitor, molecular docking, MM-PBSA, MD simulations, ADMET study, SAR

## Abstract

Benzofuran and 1,3,4-oxadiazole are privileged and versatile heterocyclic pharmacophores which display a broad spectrum of biological and pharmacological therapeutic potential against a wide variety of diseases. This article reports in silico CADD (computer-aided drug design) and molecular hybridization approaches for the evaluation of the chemotherapeutic efficacy of 16 S-linked *N*-phenyl acetamide moiety containing benzofuran-1,3,4-oxadiazole scaffolds **BF1–BF16**. This virtual screening was carried out to discover and assess the chemotherapeutic efficacy of **BF1–BF16** structural motifs as *Mycobacterium tuberculosis* polyketide synthase 13 (Mtb Pks13) enzyme inhibitors. The CADD study results revealed that the benzofuran clubbed oxadiazole derivatives **BF3**, **BF4**, and **BF8** showed excellent and remarkably significant binding energies against the Mtb Pks13 enzyme comparable with the standard benzofuran-based **TAM-16** inhibitor. The best binding affinity scores were displayed by 1,3,4-oxadiazoles-based benzofuran scaffolds **BF3** (−14.23 kcal/mol), **BF4** (−14.82 kcal/mol), and **BF8** (−14.11 kcal/mol), in comparison to the binding affinity score of the standard reference **TAM-16** drug (−14.61 kcal/mol). 2,5-Dimethoxy moiety-based bromobenzofuran-oxadiazole derivative **BF4** demonstrated the highest binding affinity score amongst the screened compounds, and was higher than the reference Pks13 inhibitor **TAM-16** drug. The bindings of these three leads **BF3**, **BF4**, and **BF8** were further confirmed by the MM-PBSA investigations in which they also exhibited strong bindings with the Pks13 of Mtb. Moreover, the stability analysis of these benzofuran-1,3,4-oxadiazoles in the active sites of the Pks13 enzyme was achieved through molecular dynamic (MD) simulations at 250 ns virtual simulation time, which indicated that these three in silico predicted bio-potent benzofuran tethered oxadiazole molecules **BF3**, **BF4**, and **BF8** demonstrated stability with the active site of the Pks13 enzyme.

## 1. Introduction

**Figure 1:**
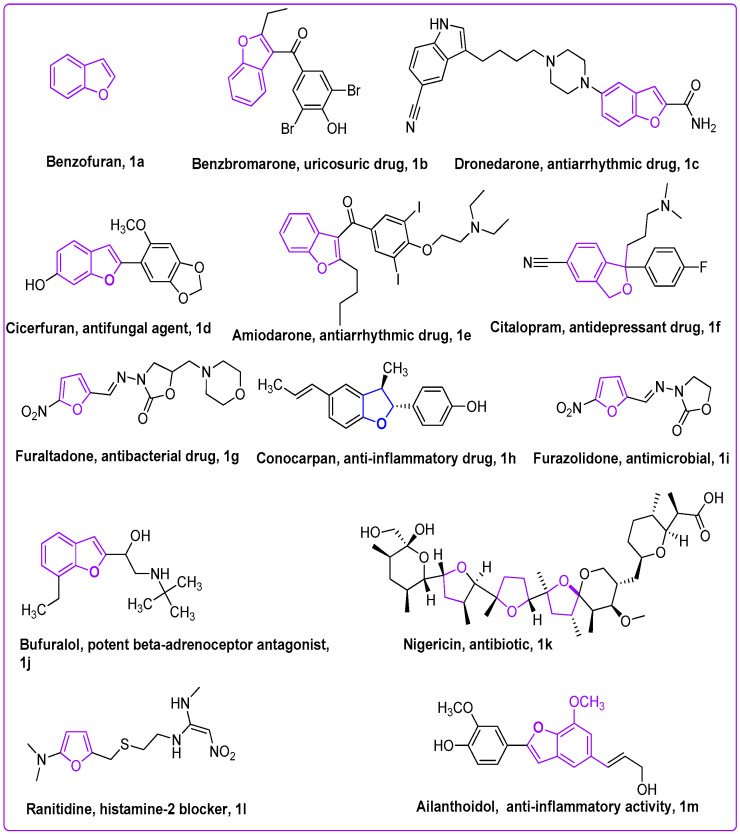
Structures of natural and synthetic furan scaffolds **1a–1m**.

**Figure 2:**
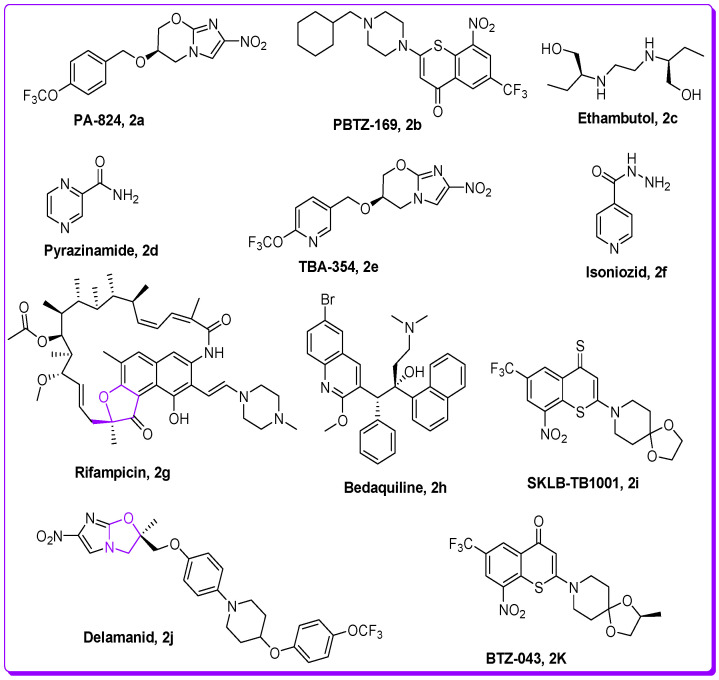
Structures of commercially available anti-TB drugs in market.

**Figure 3:**
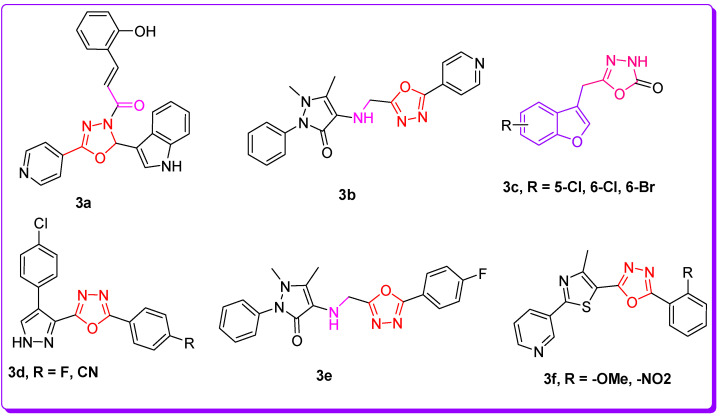
Structures of oxadiazole and benzofuran-based anti-TB scaffolds.

TB (tuberculosis) is among the deadliest transmittable infectious lung disease caused by *Mycobacterium tuberculosis* (Mtb). The global TB report 2018 described that Mtb is the leading cause of single infectious disease [1]. TB and its percentage are higher than human immunodeficiency virus (HIV) and AIDS. Amongst the infectious diseases after HIV, TB is a global health issue, being the second major cause of death across the world. In today’s world, antibiotic resistance is a major global problem in curing contagious microbial diseases (CMD) caused by deadly microbes [2,3]. The drug-resistant TB is a major health concern of today’s scientific community due to its resistance against the first-line drug rifampicin (RR-TB) [4,5]. To counter multi-drug resistance (MDR) and pan-drug resistance bacteria resulting from the development of mutagenicity [4,5,6,7,8], novel wide spectrum chemotherapeutic agents are urgently necessitated. It is imperative to discover novel anti-tuberculosis chemotherapeutic agents to stop resistance in Mtb strains, and ideally cure the disease in a shorter time [4,5,6,7,8,9,10]. There is an urgent demand and necessity for the designing, discovery, and development of novel curative candidates active against all forms of Mtb [8,9,10,11,12,13]. Heterocycles-based ring systems such as quinoxalines, coumarins, benzothiazoles, benzoxazoles, thiadiazoles, benzofurans, and oxadiazoles, etc., constitute a powerful backbone of different chemotherapeutic agents with a wide spectrum of biological activities in medicine, pharmacology, and pharmaceutics [14,15,16,17,18,19,20,21]. Benzofuran (Figure 1) is a five-membered oxygen containing a fused heterocyclic compound first synthesized by Perkin in 1870 [22]. Benzofuran moiety is an important structural unit of many of the natural and synthetic derivatives (Figure 1) displaying a versatile array of biological activities against a wide variety of diseases, such as anti-cancer, hemolytic, thrombolytic [23], anti-microbial [24], anti-tuberculosis [25,26], anti-Alzheimer’s [27,28], inflammation inhibitory activity [29], anti-parasitic [30], anti-viral [31], analgesic, anti-pyretic [32], anti-bacterial [33], anti-hyperglycemic [34], and anti-oxidant activities [33,35]. On the other hand, 1,3,4-oxadiazole scaffolds showed a broad spectrum of pharmacological applications; for example, anti-Alzheimer’s [36], anti-neoplastic [37], anti-viral [38], anti-cancer [39], FAK inhibitors [40], anti-fungal [41], anti-inflammatory [42], anti-bacterial [43] and anti-tubular activities [44]. The benzofuran-oxadiazoles also exhibited anti-lung cancer, human tyrosinase inhibitors, anti-HepG-2, anti-hemolytic, and anti-thrombotic activities [24,45]. The different marketed anti-TB drugs are listed in Figure 2, while potent anti-TB bioactive compounds with oxadiazole and furan cores are depicted in Figure 3 [46,47,48,49,50,51,52].

**Figure 4:**
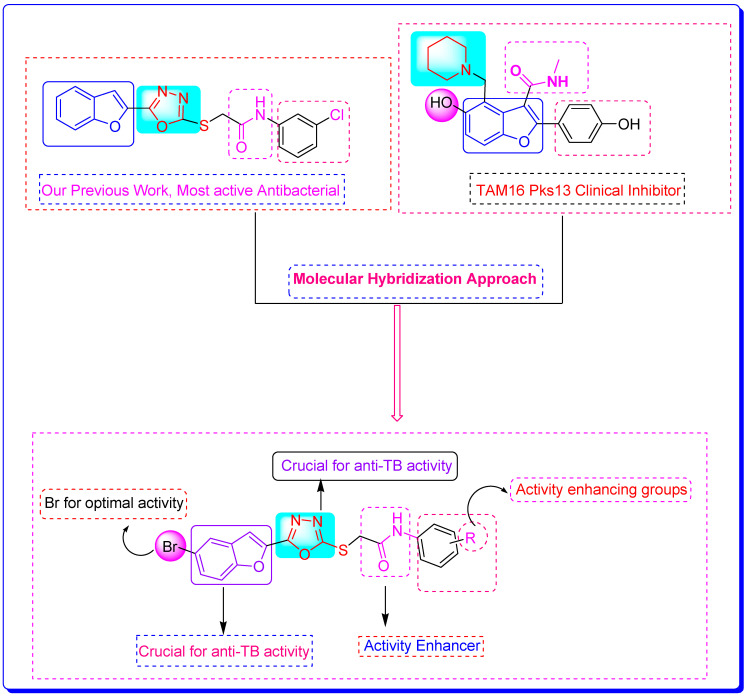
Rational design of benzofuran-1,3,4-oxadiazole derivatives **BF1–BF16** as anti-tuberculosis agents [24,46,47].

The previous work reported on benzofuran-oxadiazoles as anti-microbial agents by our research group and the current comprehensive literature study (Figure 4) [17,42,43,44,45,46,47,48,49,50,51,52] is the basis of the rationale to discover novel benzofuran-appended oxadiazole structural hybrids as anti-TB drug candidates with the help of different in silico techniques.

## 2. Results and Discussion

### 2.1. Evalaution of Anti-Mtb Potenial of Benzofurans-1,3,4-Oxadiazoles Using Computational Approaches

#### Mycobacterium Tuberculosis

Benzofuran-1,3,4-oxadiazoles **BF1–BF16** [23,45,52] were evaluated for their anti-Mtb potential using computational approaches. Tuberculosis is a bacterial disease that predominantly affects the lungs and is caused by *Mycobacterium tuberculosis* [53]. Even though macrophages are crucial to the host immune system because they identify and eliminate potential intruders (pathogens), Mtb has developed several tactics that allow it to live and proliferate inside these lung macrophages, which are the main host cells for Mtb infection [54]. It is one of the prevalent causes of death worldwide, particularly in people who have also been infected by other pathogenic viruses and have viral infections. The existing TB treatment regimens take between six and nine months to complete, which makes it difficult for patients to adhere to them, which causes multi-drug resistant TB, which is essentially resistant to antibacterial drug treatments [55]. Hence, new potential anti-TB chemotherapeutics need to be designed and developed to overcome the MDR problem by targeting novel pathways and enzymes involved in bacterial growth, with new modes of action to lessen the likelihood of a relapse of the TB infection when existing medications are no longer reliable [5,6,13].

Intensive research has been put into finding new Mtb targets and their inhibitors that can stop the spread of this bacterial pathogen and mitigate its drug resistance [6,13,56]. Similarly, an important class of enzymes known as polyketide synthases (Pks) has not been explored as a therapeutic target for microbial infection. The Pks are enzymes that produce mycolic acids, which are essential for the survival and pathogenicity of Mtb [57]. More than 20 (Pks) enzymes are a part of various multi-enzyme complexes that work together to produce mycolic acid in Mtb [58]. These Pks13-derived lipid metabolites (mycolic acids) are important constituents of Mtb’s distinctively sophisticated and lipid-rich cell wall [59], which has been suggested as a way for it to live in hostile environments in host macrophages while also providing an inherent resistance to numerous anti-microbial medications [60]. As it is recognized that these mycolic acids, a feature of the genus mycobacterium, are essential for the survival of this pathogen, disruption of this important biosynthesis pathway is a promising drug target for TB mitigation [59,60].

One of the important enzymes of the Pks enzyme family is Pks13, which is responsible for the condensation of two fatty acid chains into α-alkyl β-ketoacyl, a primary precursor of the mycolates. Therefore, Pks13 is a crucial enzyme for mycobacterial survival, making it a desirable new target for the pursuit of possible anti-tuberculosis drug candidates [61]. Research has been going on to identify drugs targeting this Pks13 enzyme, and several benzofuran-based scaffold-carrying compounds have been identified as potent repressive agents of Pks13 using structure-based drug design techniques [17,42,43,62]. These novel benzofurans oxadiazoles **BF1–16** had strong anti-Pks13 activity in various investigational models [46,63]. Moreover, benzofuran-based compounds have also been identified to target other vital enzymes of Mtb [64]. Taking into account these potent inhibitory activities of benzofuran-based compounds against *Mycobacterium tuberculosis*, we will evaluate the potential anti-Mtb activities of our synthesized compounds (**BF1** to **BF16**) in this study by utilizing computer-aided drug discovery techniques (CADD) by targeting the important Pks13 enzyme of Mtb.

### 2.2. Molecular Docking Investigations of BF1–BF16 against the Pks13 Enzyme

**Figure 5:**
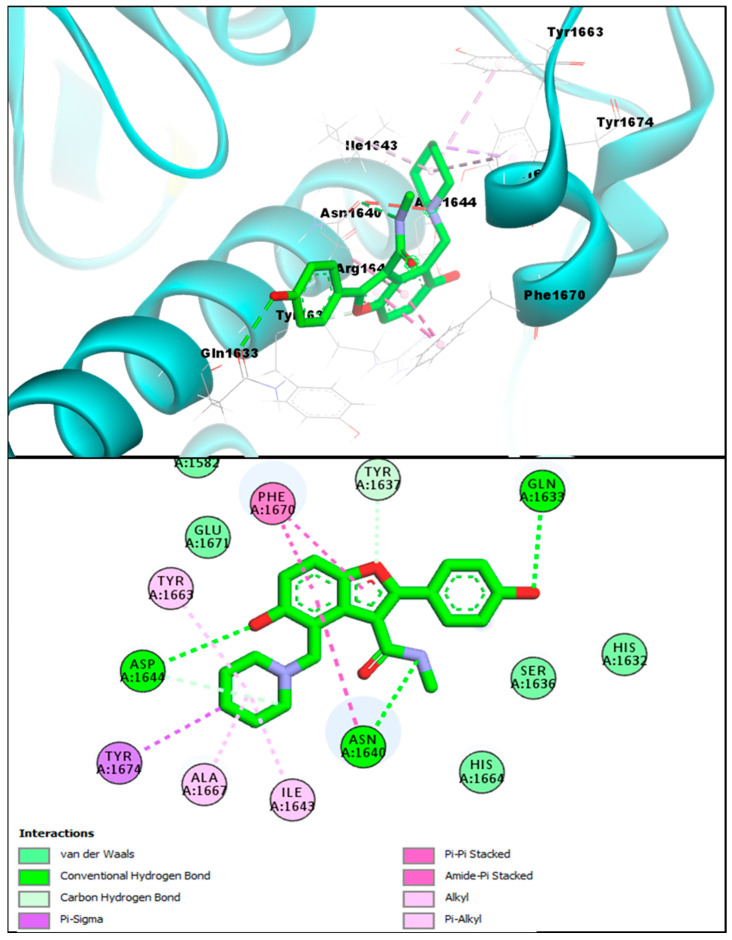
**TAM-16** inhibitor (upper panel) 3D pose (obtained through molecular docking) with the Pks13 interacting residues labeled, and its 2D interactive pose (lower panel) with the Pks13 enzyme.

The in silico molecular docking approach was utilized to screen the 1,3,4-oxadiazole based benzofuran compounds via MOE (molecular operating environment) against the Pks13 of Mtb, and these results were compared with the co-crystallized inhibitor (TAM-16) of Pks13.The standard reference benzofuran **TAM-16** inhibitor of anti-Mtb displayed an excellent binding affinity score (−14.61 kcal/mol) due to its strong binding interaction with the Pks13 enzyme active site (which is involved in the Mtb mycolic acid biosynthesis). The conformation analysis of the interaction of **TAM-16** inhibitor with the active site pocket of Pks13 indicated that the **TAM-16** inhibitor interacts with multiple amino acids residues (ASP1644, ASN1640, and GLN1633). The **TAM-16** inhibitor made different multiple conventional and carbon-hydrogen-type hydrogen bonds with different amino acids of the active site. In the analysis, the Pks13 and **TAM-16** protein-ligand complex demonstrated several other Pi-Pi and Amide-Pi Stacked-type molecular interactions. In addition, other types of molecular interactions—such as Alkyl, Pi-Alkyl, and Pi-Sigma interactions, which stabilize a compound inside a pocket—were also observed with the ILE1643, TYR1663, ALA1667, and TYR1674 between the Pks13 and the **TAM-16** inhibitor. Furthermore, researchers that first discovered **TAM-16** against Pks13 have stated that it exerts its inhibitory action by obstructing access to the Pks13 active site, which houses the catalytic triad (Ser1533, Asp1560, and His1699); in this study, it can be seen that **TAM-16** also occupied the active site pocket and blocked access to these residues, as seen in Figure 5.

**Figure 6:**
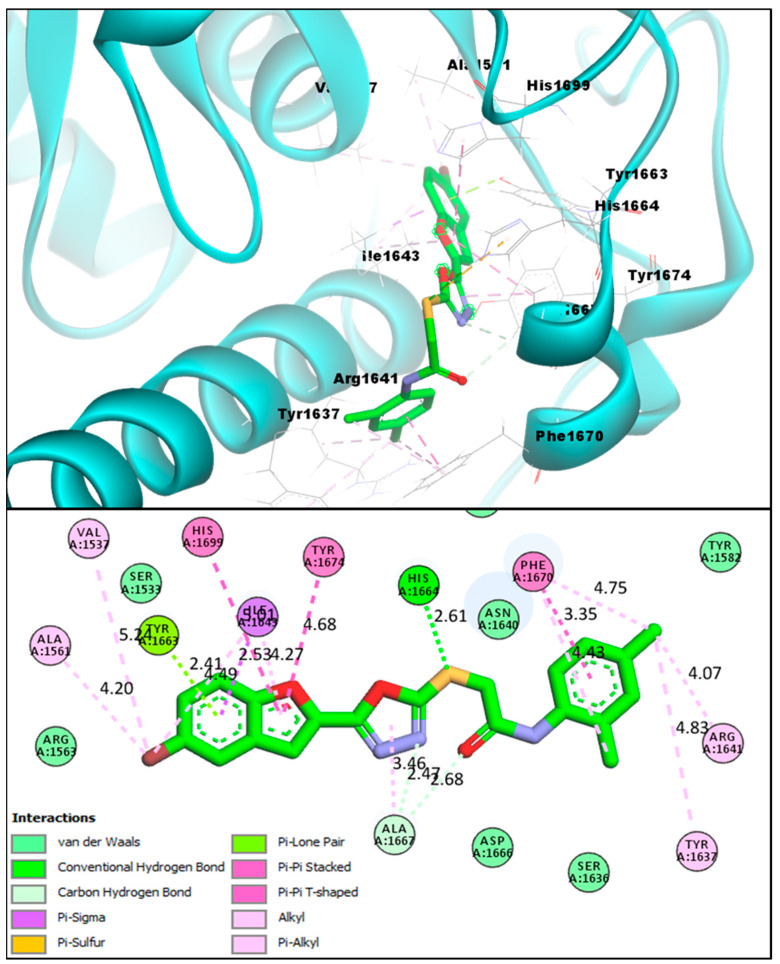
**BF3** compound’s 3D pose (upper panel) interacting receptor residues are labeled, and its 2D interactive pose (lower panel) with the Pks13 enzyme.

The afforded benzofuran molecules demonstrated strong binding affinities and robust interactions with the Pks13 enzyme active site in comparison to the benzofuran-based **TAM-16** reference standard inhibitor. Out of the 16 novel synthesized analogs **BF1** to **BF16**, three of them, **BF3**, **BF4**, and **BF8** showed similar affinities to that of **TAM-16** with the Mtb Pks13 enzyme. The conformation analysis of the pose and binding affinity of benzofuran-appended 1,3,4-oxadiazole derivative **BF3** showed the binding affinity score (−14.23 kcal/mol) with the Pks13 active site due to the multiple molecular interactions with the Pks13 receptor residues. The benzofuran ring of the derivative **BF3** demonstrated hydrogen bonds of conventional and carbon-hydrogen types with TYR1663, along with an H-bond between the HIS1664 and the sulphur atom. Moreover, other interactions such as Pi-Pi T-shaped (With the HIS1699 catalytic residue), Pi-Pi Stacked, Pi-Sigma and Alkyl were present, as were Pi-Alkyl interactions between the benzofuran ring and the Pks13. Several of the Pks13 receptor residues also made Van der Waals and Pi-Lone Pair molecular interactions with this compound; they are graphically presented in Figure 6.

**Figure 7:**
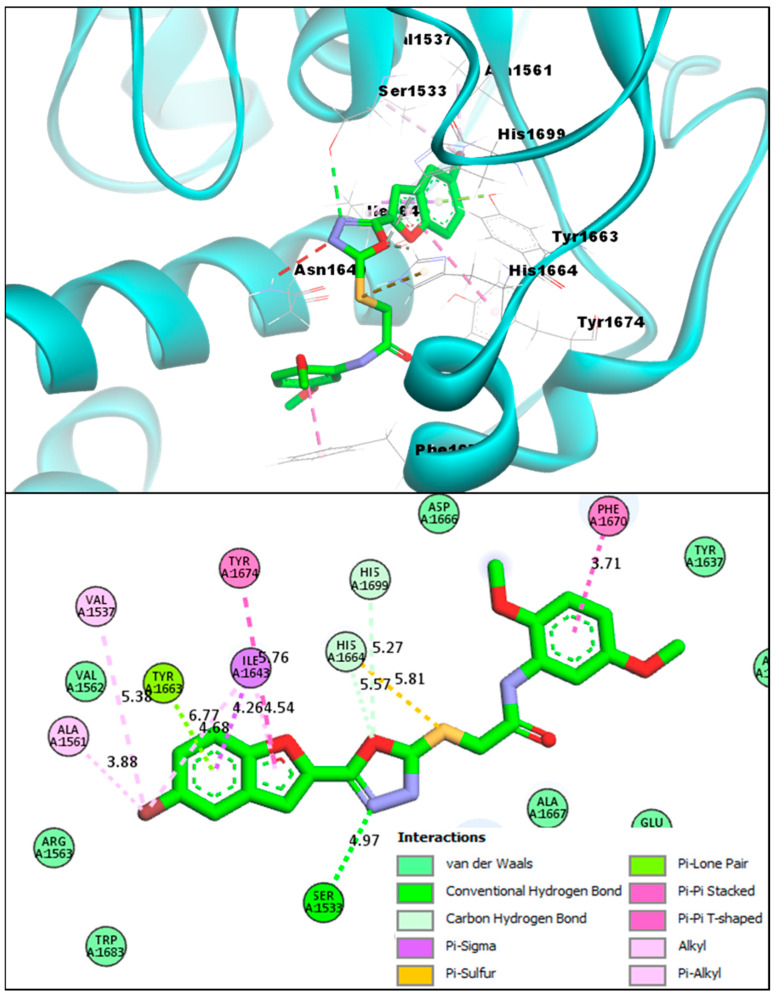
**BF4** compound’s 3D pose (upper panel) interacting receptor residues are labeled, and its 2D interactive pose (lower panel) with the Pks13 enzyme.

During the molecular docking studies, compound **BF4** also showed a strong binding affinity score with the Pks13 active site (−14.82 kcal/mol) as compared to the **TAM-16** binding affinity, which was (−14.61 kcal/mol) with the Pks13 enzyme; **BF4** showed relatively stronger binding to the active site of the target protein. Furthermore, the interaction analysis of **BF4** and the Pks13 protein complexes showed that **BF4** directly interacted with the catalytic residues (Ser1533 and His1699), which are directly involved in the mycolic acid synthesis and are essential to the Pks13 activity. The benzofuran **BF4** blocked access to these catalytic residues by interacting similarly to the **TAM-16** inhibitor, as mentioned in previous paragraphs. It can be seen in Figure 7 that **BF4** was able to engage the HIS1699 catalytic residue via a carbon-hydrogen-type hydrogen bonding with the oxygen atom of the oxadiazole ring. This oxadiazole ring’s nitrogen atom also engaged the other important catalytic residue (SER1533) by forming a conventional hydrogen bond. Furthermore, the benzofuran moiety of **BF4** also made multiple molecular contacts, as was previously seen in the **BF3** and Pks13 complex, and formed a hydrogen bond with the TYR1663, a Pi-Sigma interaction with the ILE1643, and multiple stabilizing interactions with Alkyl, Pi-Alkyl, Pi-Pi T-shaped and Pi-Pi Stacked; these Pi-Sulfur and Pi-Sigma interactions were also present in **BF4** and the Pks13 complex.

**Figure 8:**
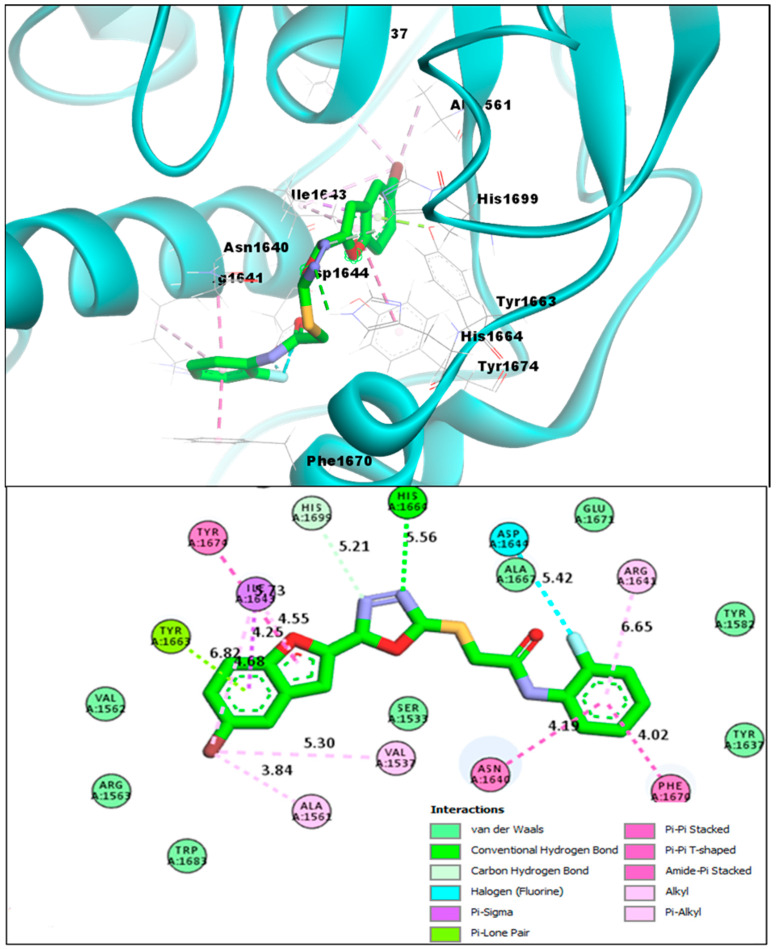
**BF8** compound’s 3D pose (upper panel), the interacting receptor residues are labeled, and its 2D interactive pose (lower panel) with the Pks13 enzyme.

The third novel benzofuran **BF8** also showed comparable binding affinity in comparison to that of **TAM-16**, and was able to bind to the Pks13 active site with a binding affinity score of −14.11 kcal/mol. It also directly engaged one of the crucial catalytic residues HIS1699 with its oxadiazole ring’s nitrogen atom via a conventional H-bond. Moreover, the benzofuran moiety of **BF8** also showed stable molecular interactions of multiple types with the Pks13 receptor residues, and the TYR1663 made an H-bond with the bromine atom of this ring and made Alkyl and Pi-Alkyl interactions, as previously seen in the other benzofurans. In addition, other molecular interactions, i.e., Pi-Pi T-shaped, Pi-Pi Stacked-type, Amide-Pi Stacked, Pi-Sigma, and interactions of fluorine on the phenyl ring of **BF8** with the ASP1644 of Pks13 receptor were also seen in **BF8** and the Pks13 protein complex, as seen in Figure 8.

An overview of the binding affinity scores of the studied bromo-substituted benzofuran-1,3,4-oxadiazoles **BF1–BF9** and the interactive residues engaged by these compounds in the active pocket of the Pks13 enzyme are presented in Table 1; while the bromo-unsubstituted benzofuran-1,3,4-oxadiazole structural hybrids **BF10–BF16** that showed less binding affinity scores with the Pks13 enzyme are presented in the Supplementary Table S1.

### 2.3. Structure-Activity Relationship (SAR) of Bromobenzofuran-1,3,4-Oxadiazoles BF3, BF4, and BF8

**Figure 9:**
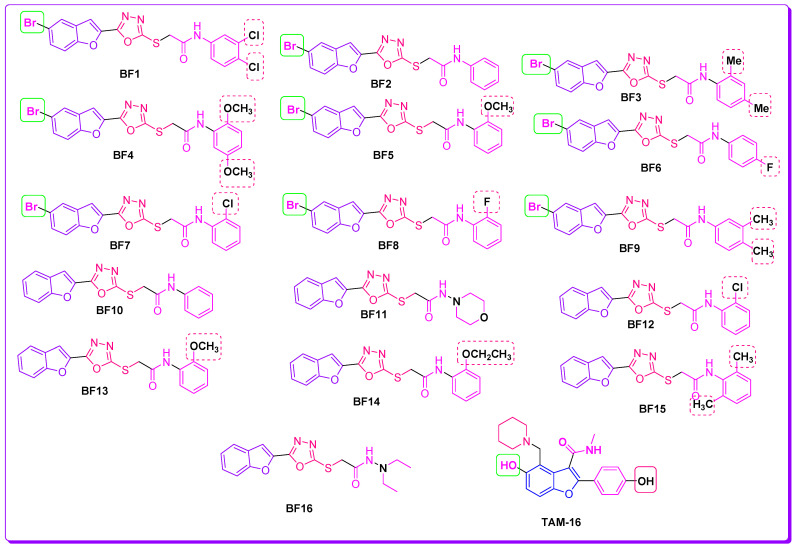
Structural data of benzofuran-1,3,4-oxadiazole derivatives **BF1–BF16 and TAM-16** inhibitor.

The analysis of the structure-activity relationship (SAR) of the novel 5-bromobenzofuran-oxadiazole compounds **BF1–BF9** revealed that the simple benzofuran moiety containing oxadiazole molecules **BF10–BF16** showed less binding affinity towards the Pks13 enzyme active site as compared to the 5-bromo moiety-based benzofuran-oxadiazoles. Among the 5-bromobenzofuran-oxadiazole compounds, **BF3** and **BF8** compounds having methyl (-CH_3_) and highly electronegative atom fluorine on the phenyl rings (Figure 9) displayed comparable binding affinities with reference to the benzofuran **TAM-16** standard Pks13 inhibitor. Meanwhile, the 2,5-dimehoxy functionality containing the **BF4** molecule demonstrated stronger binding affinities due to stable conformation, alignments, robust interactions, and direct bindings to the catalytic residues of Pks13 active site pocket compared to the standard **TAM-16** inhibitor, which only blocked access to the Pks13 of Mtb active site pocket residues. Overall, the SAR study of all **B1–BF9** predicted that phenyl position 2 of the *N*-phenyl acetamide fragment is more active in comparison to other positions in the S-alkylated 5-bromobenzofuran-oxadiazoles tethered *N*-phenyl acetamides **BF1–BF9**. These findings suggest that the in silico identified compounds can be effective novel anti-Mtb agents, and may help tackle the resistant strains of Mtb and reduce the treatment times if used in combinatorial therapy against *Mycobacterium tuberculosis* infections.

### 2.4. ADMET and Drug-Likeness Studies of Benzofuran-1,3,4-Oxadiazoles BF1–BF16

The ADMET and drug likeness studies of novel benzofuran clubbed 1,3,4-oxadiazole compounds **BF1–BF9** are already reported by Irfan et al. [23,45,52]). In this study, ADMET and drug likeness studies of benzofuran oxadiazoles **BF10–BF16** (Supplementary Tables S2 and S3) were carried out in order to check their profile in comparison to previously reported derivatives **BF1–BF9**. In general, benzofuran oxadiazoles **BF1–BF16** showed good human intestinal absorptions and were classified as HIA+ based on pharmacokinetics and ADMET analysis. The novel benzofuran-appended 1,3,4-oxadiazoles demonstrated acceptable lipophilic (iLogP) characteristics and good Log S (ESOL) water solubility values. Additionally, they were not P-gp protein substrates (P-glycoprotein, or P-gp, is a transporter protein of cell membranes that controls the efflux of substances and medications from cells). Studies on metabolism have revealed that these substances are CYP450 3A4 substrates, which means that after these drug-like compounds have completed their task inside the body, the CYP450 3A4 can easily biotransform these substances inside the liver before sending them to the excretory organs for excretion from the body. These novel compounds were also non-inhibitors of the renal-OCT proteins (transporter proteins), which are crucial in the detoxifying/excretion of exogenous chemicals/drugs from the body. The toxicity investigations of these substances also revealed that they are not carcinogenic, non-AMES toxic, do not affect or inhibit the ThERG II ion channel that regulates cardiac action potential repolarization, and are non-interferers in its regular operation. According to these studies, just like **BF1–BF9**, the derivatives under study **BF10–BF16** had shown favorable ADMET properties compared to the standard **TAM-16**, which shows that these compounds, if utilized, would pose no significant health hazards to its subjects upon administration.

Moreover, the drug-likeness investigations involving identifying these compounds’ physicochemical properties and medicinal chemistry showed that these **BF1–BF9** and **BF10–BF16** compounds had an excellent topological surface area (TPSA), acceptable molecular weight values, and good synthetic accessibility scores. These benzofuran **BF1–BF9** and **BF10–BF16** compounds followed the drug-likeness rules, such as Pfizer and Lipinski’s drug rules. These compounds also showed no PAINS alerts, and along with this, they also followed the Golden Triangle rule and had good bioavailability scores (greater than 0.10). ADMET and drug-likeness data of **BF10–BF16** along with the reference compound **TAM-16** are presented in the Supplementary Tables S2 and S3, respectively.

### 2.5. MD Simulations Study of Benzofuran-1,3,4-oxadiazoles BF3, BF4, and BF8

**Figure 10:**
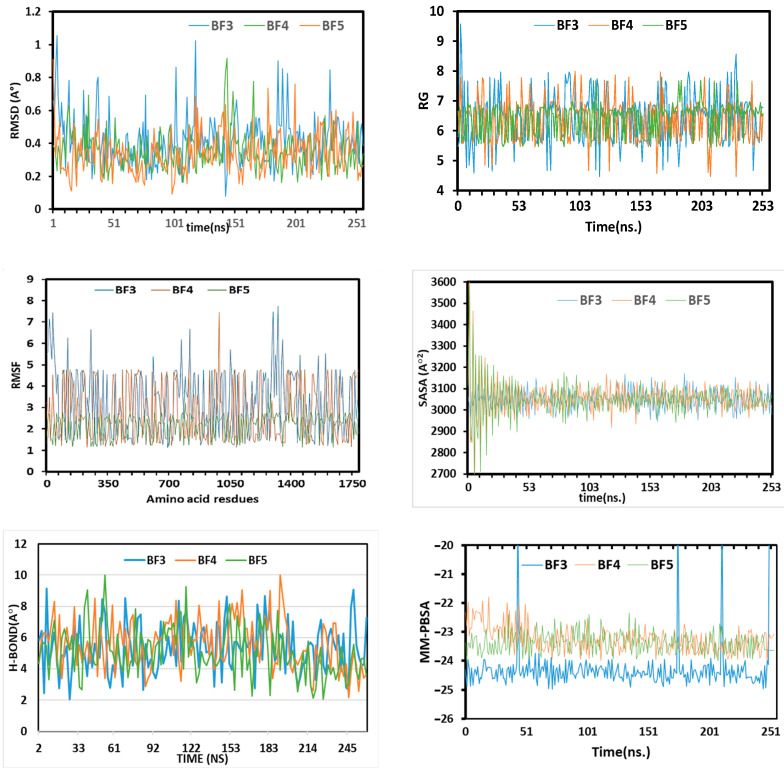
The molecular dynamics simulation trajectories from 250-ns simulation time.

The variation and stability of the in silico predicted bioactive benzofuran-1,3,4-oxadiazoles **BF3**, **BF4**, and **BF8** were studied by applying the C-alpha atoms root means square deviation (RMSD) approach. The three complexes displayed the initial upper phase, which resulted in the flexible behavior depicted in Figure 10. At the 25 ns simulation time, the complexes reached a steady state and the complexes retained stability until the simulations’ last segment. On the other hand, **BF3** had fluctuations at 150 ns, 180 ns, and 230 ns. The higher RMSD for **BF3** than other compounds demonstrates how these complexes are more adaptable than the other molecules, **BF4** and **BF8**. The analyzed complexes were defined as having a stable comparative characteristic because their RMSD was below 2.5 during the simulation. The SASA simulation (solvent-accessible-surface-area) was carried out on complexes **BF3**, **BF4**, and **BF8**. The SASA simulation approach was utilized to study the variation of the complexes’ topology, which showed that higher SASA reflected the extent of the surface volumes, while the elongated nature defined the lower SASA, as shown in Figure 9. Their stability was proved by the steady-state for **BF3**, **BF4**, and **BF8** after 48 ns and the low fluctuation degree for SASA profiles along the simulated trajectories. Additionally, each complex had SASA degrees that were roughly comparable, and the interaction over **BF3**, **BF4**, and **BF8** compounds described these protein complexes in their compact form. The **BF3**, **BF4**, and **BF8** complexes were analyzed for radius-gyration (RG) and trajectories. The trajectories demonstrated the flexibility and degree of mobility. The complexes **BF3**, **BF4**, and **BF8** RG steady degree appeared at 7 ns.

To assess the flexibility of the residues of amino acids, the root-means-square-fluctuations (RMSF) were likewise also explored (Figure 10). The high Firmness of **BF4** and **BF8** complexes was demonstrated by their low RMSF values of 2.5 Å, but the relative flexibility of the back-bone of amino acid residues in the **BF3** complex was demonstrated by its growing RMSF value of 6Å. Additionally, the small variations in the RMSD of **BF3**, **BF4**, and **BF8** systems demonstrated loops variations in Mtb Pks13, which are naturally flexible. These RMSD variations correspond to enzyme structure adaptations in order to strongly engage the compounds at the binding site, as suggested by the H-bonding pattern. [65,66].

The H-bond played an important role in identifying the stability of the interaction-strength in the ligand and protein. The in silico predicted bioactive **BF3**, **BF4**, and **BF8** have constant H-bonds range between 2 and 10 in the simulation process. The changing H-bond between the ligand-enzyme may suggest that the conformation around the ligands inside the binding site change through simulation. Overall, simulations supported the high stability of all protein-ligand complexes.

### 2.6. MM-PBSA Investigations of the Most In Silico Bioactive Benzofuran-1,3,4-oxadiazoles

**Figure 11:**
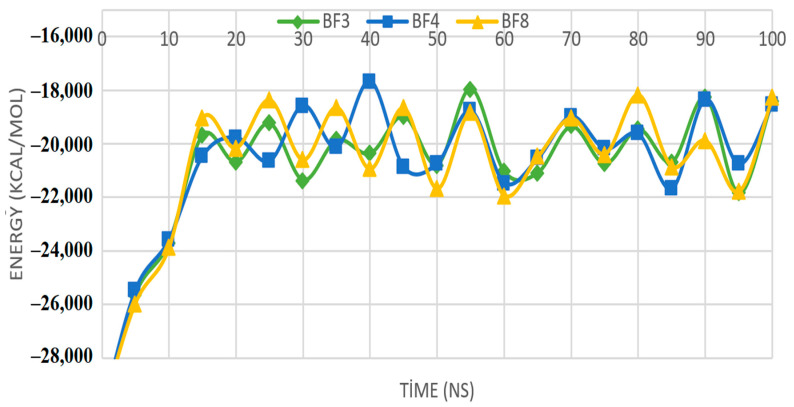
Display of Gibbs free energy values of protein and ligand molecules at ten ns intervals.

Deciphering intermolecular interactions and energies at different nanoseconds are important to unveil microscopic information important for guiding stable docked complexes. This in turn ensures the selection of compounds that can inhibit the receptor enzyme. Although the ligand molecules are pretty flexible in the calculations, the Pks13 protein does not have this flexibility. In Molecular Mechanics-Poisson-Boltzmann surface area (MM-PSBA) calculations, the interaction between the ligand and Pks13 protein is done at the ns level. Both the ligand molecule and the Pks13 protein have flexibility in calculations of MM-PSBA. The interaction occurring in these calculations were examined every 10 ns, and the energy change is given in Figure 11.

The generated graph displays the binding free energy variations and changes for each interval of ten (ns), along with standard deviations (±) given in Table 2. The high affinity bromobenzofuran-1,3,4-oxadiazole binders to Mtb Pks13 were **BF3**, **BF4**, and **BF8** molecules, which were then compared to one another. Using this comparison, calculations were performed to support the (MM-PBSA) method’s estimation of the bonding’s free energies. The relevant parameters’ negative values signify stronger binding [67]. According to calculations using equation-1, the average values of Gibbs free energies are −31.4 kcal/mol for Pks13+**BF3**, −48.8 for Pks13+**BF4**, and −41.5 for Pks13+**BF8**. It can be seen in Table 2 that at each ns, the compounds showed robust strong intermolecular interactions energy. This further demonstrates the formation of high stable complexes and the strong binding of the compounds to the Mtb Pks13 enzyme. However, as standard deviation values are moderate to high, further extensive calculations are needed to validate the energy values. In light of these results, it can be seen that the free energy values of these three molecules show that they may possess better repressive properties against the important Mtb Pks13 enzyme.

## 3. Materials and Methods

### 3.1. Chemistry

All the benzofuran-oxadiazole structural motifs **BF1–16** were afforded, and their characterization data were published by Irfan et al. [23,45,52]. The structures of benzofuran-1,3,4-oxadiazoles **BF1–BF16** along with the **TAM-16** standard reference inhibitor structure are given in Figure 11.

### 3.2. Molecular Docking of Benzofuran-1,3,4-oxadiazoles BF1–BF16

The protein PDB structure of the target enzyme Pks13 of *Mycobacterium tuberculosis* was achieved from the RCSB to carry out the molecular docking study (computational research) [68] website with the PDB Identifier (5V3Y) [62]. The molecular docking study of sixteen novel benzofuran-1,3,4-oxadiazoles was carried out with MOE (Ver-2009.10) software [69]. The first step was the preparation of protein structure of the Pks13 enzyme by removing water molecules and heteroatoms from the protein PDB structure with the help of Biovia DS [70] software for molecular docking study. The Chem-draw professional (Ver-16) software [71] (by PerkinElmer Informatics) was used to draw the structures of benzofuran-1,3,4-oxadiazole ligands **BF1–BF16** and saved in the mol format for further studies. Before docking, the ligand structures were loaded in the MOE, and their energy was minimized via the MMFF94x forcefield, and the partial charges were also added to the ligand structures. Using the Triangle Matcher placement strategies, the compounds were docked in the binding pocket and scored by the Dock module using the London-dG scoring function of MOE. The protein PDB was opened in MOE, and was 3D-protonated using the Amber99-ff. The active site of the Pks13 was located and selected using the Site Finder function of MOE. The ligand-protein interaction was viewed using the software Biovia DS Studio (Ver-2017) [72].

### 3.3. ADMET and Drug-Likeness Investigations of Benzofuran-1,3,4-oxadiazoles

The ADME and drug-likeness studies of benzofuran-1,3,4-oxadiaole compounds were carried out by utilizing the Swissadme (Ver-1) [73] and ADMET lab (Ver-2.0) [74] online web-servers, while for the toxicity investigations, the ADMETSAR (Ver-1.0) [75,76] online server was utilized.

### 3.4. MD Simulation Study of the Most In Silico Bioactive BF3, BF4, and BF8 Derivatives

The MD Simulations of the most in silico predicted biologically active benzofuran-1,3,4-oxadiazole **BF3**, **BF4**, and **BF8** scaffolds were performed by GROMACS. Using GROMACS (Ver-2021) and the Linux 5.4 package, MD simulation of the protein-ligand complexes was carried out. The ligand topologies were created using the PRODRG server, and the GROMOS96 forcefield was used as the force field for proteins. Simple point charge (SPC) water molecules in a rectangular box were used to solvate each complex. Na + and Cl + ions were added to electrically make the simulation system neutral, whereas salt concentrations of 0.15 mol/L were set in each system. All solvated complexes underwent energy minimization for 5000 steps using the steepest descent method. Different productions were run in the MD simulation, including the constant number of particle, pressure, and temperature (NPT) series and the constant number of particle, volume, and temperature (NVT) series. For the simulation, a V-rescale thermostat and a Parrinello-Rahman barostat were chosen, and the NVT and NPT series were conducted at 300 K and 1 atm for 300 ps. Finally, the production run was completed after 250 ns at 300 K. [77,78].

### 3.5. MM-PBSA Binding Free Energy Calculations of the Most In Silico Bioactive BF3, BF4, and BF8 Derivatives

Molecular mechanics Poisson-Boltzmann surface area (MM-PBSA) calculations of molecules were made with molecular dynamic calculations. The different types of binding free energies, i.e., Van der Waals, electrostatic, kinetic, and potential energy changes of the studied **BF3**, **BF4**, and **BF8** molecules were determined for these calculations. In addition, this study examined the interactions between **BF3**, **BF4**, and **BF8** molecules and the 5V3Y protein, which is the Pks13 protein. These interaction energies were investigated at 100 ns. As a result of the interaction of the three molecules studied with the protein, the values of the binding free energy change were calculated. This calculation is given in Equation (1).

(1) $$\Delta G_{Bind}=G_{complex}-\left(G_{res}+G_{lig}\right)$$

In the above equation, $\Delta G_{Bind}$ gives the total binding free energy value between the ligand and the Pks13 protein. $G_{lig}$, $G_{res}$, and $G_{complex}$ values in the equation are the values of the ligand molecule, Pks13 receptor protein, and complex molecule, respectively [79].

## 4. Conclusions

In conclusion, the novel series of sixteen benzofuran-1,3,4-oxadiazoles **BF1–BF16** was evaluated for their therapeutic inhibitory effect on Mtb Pks13 enzyme by applying various in silico approaches, such as molecular docking, MM-PBSA, pharmacokinetics, ADMET, and molecular dynamic simulations. The results of the CADD approach indicated that three in silico predicted lead compounds such as the 2,4-dimethyl moiety containing **BF3**, 2,5-dimethoxy moiety-based **BF4**, and 2-flouro moiety containing **BF8** displayed excellent in silico anti-TB chemotherapeutic potential due to the strong interaction with the active site of the Pks13 enzyme and the greater stability of these complexes in comparison to the standard reference benzofuran drug **TAM-16.** The conformational pose and binding affinity analysis of **BF3**, **BF4**, and **BF8** showed that these derivatives bind to the Pks13 active site with a binding affinity score of −14.23 kcal/mol, −14.82 kcal/mol, and of −14.11 kcal/mol, respectively, in comparison with the standard reference **TAM-16** (−14.61 kcal/mol). The **BF4** bromobenzofuran-1,3,4-oxadiazole showed higher binding affinity −14.82 kcal/mol than the reference Pks13 inhibitor **TAM-16** (−14.61 kcal/mol), which was further confirmed by the MM-PBSA calculations and the MD simulations studies. The ADMET studies of all the screened bromobenzofuran-oxadiazole structural hybrids demonstrated a high degree of drug-likeness profile. Overall, it is seen that the bromobenzofuran-1,3,4-oxadiazole **BF4** derivative has a more stable total binding free energy value against the 5V3Y protein. On the basis of different in silico techniques, 2,5-dimethoxy phenyl-substituted bromobenzofuran-1,3,4-oxdiazole **BF4** is a more in silico predicted effective reagent than **TAM-16**, so this in silico bioactive **BF4** can be a future lead anti-TB chemotherapeutic candidate after further in vitro and in vivo evaluations, which would be necessary to establish its chemotherapeutic efficacy against Mtb.

## Figures and Tables

**Table 1:** The molecular docking profile of bromobenzofuran-oxadiazoles **BF1–BF9** against Pks13.

Compounds	Binding Affinities	Interacting Residues of Pks13	Interaction Types
**BF1**	−12.93 kcal/mol	ILE1643, TYR1663, HIS1632, HIS1699, ALA1667, TYR1674,	Carbon-Hydrogen Bond, Van der Waals, Pi-Pi T-Shaped, Pi-Alkyl, and Alkyl.
**BF2**	−12.71 kcal/mol	ASN1640, ILE1643, TYR1637, TYR1663, HIS1632, ALA1667, TYR1674, PHE1670	C-Hydrogen Bond, Van der Waals, Pi-Pi T-Shaped, Pi-Pi Stacked, and Alkyl.
**BF3**	−14.23 kcal/mol	VAL1537, ALA1561, PHE1637, ARG1641, ILE1643, TYR1663, HIS1664, ALA1667, PHE1670, TYR1674, HIS1699	Conventional H-bond, C-Hydrogen Bond, Van der Waals, Pi-Pi T-Shaped, Pi-Alkyl, Pi-Lone pair, Pi-Sulfur, Pi-Sigma, and Pi-Pi Stacked
**BF4**	−14.82 kcal/mol	VAL1537, SER1533, ALA1561, VAL1537, TYR1674, ILE1643, PHE1670, ALA1667	Conventional H-bond, C-Hydrogen Bond, Van der Waals, Pi-Pi T-Shaped, Pi-Alkyl, Pi-Lone pair, Pi-Sulfur, Pi-Sigma, and Pi-Pi Stacked
**BF5**	−12.31 kcal/mol	ALA1561, TYR1663, ILE1643, HIS1664, TYR1674, ALA1667	C-Hydrogen Bond, Van der Waals, Pi-Pi T-Shaped, Pi-Alkyl, Pi-Lone pair, and Alkyl.
**BF6**	−11.89 kcal/mol	SER1533, ALA1667, ALA1561, TYR1663, ILE1643, HIS1664, GLN1633, TYR1674	Conventional H-bond, C-Hydrogen Bond, Van der Waals, Pi-Pi T-Shaped, Pi-Alkyl, Pi-Lone pair, Halogen, and Alkyl.
**BF7**	−12.23 kcal/mol	HIS1632, TYR1637, ILE1643, TYR1663, ALA1667, PHE1670, TYR1674	Carbon-Hydrogen Bond, Van der Waals, Pi-Pi Stacked, Pi-Alkyl, and Alkyl.
**BF8**	−14.11 kcal/mol	VAL1537, ALA1561, TYR1663, ASN1640, ILE1643, PHE1670, ARG1641, ASP1644, HIS1664	Conventional H-bond, C-Hydrogen Bond, Van der Waals, Pi-Pi T-Shaped, Pi-Alkyl, Pi-Lone pair, Amide-Pi Stacked
**BF9**	−13.44 kcal/mol	ILE1643, ALA1667, PHE1670, VAL1562, HIS1699, TYR1674, TYR1637	Conventional H-bond, C-Hydrogen Bond, Van der Waals, Pi-Pi T-Shaped, Pi-Pi Stacked, Pi-Alkyl, and Alkyl
**TAM-16 (Standard)**	−14.61 kcal/mol	SER1533, GLN1633, ASN1640, ASP1644, ILE1643, TYR1663, ALA1667, PHE1670, TYR1674	Conventional H-bond, C-Hydrogen Bond, Van der Waals, Pi-Pi Stacked, Pi-Alkyl, Amide Pi-Stacked, Pi-Sigma, and Alkyl

**Table 2:** The binding free energy changes and deviations in each ten (ns) interval. The energy values are presented in kcal/mol.

Nanoseconds	Pks13+BF3	Pks13+BF4	Pks13+BF8
10	−59.4 ± 149.6	−956.2 ± 586.2	−87.8 ± 235.6
20	−135.2 ± 235.5	−105.3 ± 387.4	−508.9 ± 245.1
30	−95.8 ± 269.3	−912.3 ± 189.3	−354.2 ± 245.3
40	−570.3 ± 684.2	−245.3 ± 245.6	−150.8 ± 250.4
50	−856.2 ± 345.6	−856.3 ± 409.8	−750.4 ± 150.6
60	−135.2 ± 248.6	−301.7 ± 204.8	−723.3 ± 523.6
70	−486.3 ± 367.3	−501.1 ± 193.5	−685.8 ± 351.2
80	−648.8 ± 385.2	−1101.3 ± 497.6	−289.7 ± 487.5
90	−329.2 ± 301.2	−687.5 ± 260.1	−350.4 ± 293.7
100	−300.8 ± 283.2	−423.4 ± 305.3	−145.8 ± 354.6

## Data Availability

Data is contained within the article and Supplementary material.

## Supplementary Materials

The following supporting information can be downloaded at: https://www.mdpi.com/article/10.3390/ph16060829/s1. Binding affinities of benzofuran-1,3,4-oxadiazole **BF10**–**16** are present in the supplementary Table S1; ADMET and drug-likeness data is depicted in Tables S2 and S3, respectively.
