# Supplementary material for: In Silico Development of Novel Benzofuran-1,3,4-Oxadiazoles as Lead Inhibitors of M. tuberculosis Polyketide Synthase 13
Source: Pharmaceuticals (Basel). 2023 Jun 1;16(6):829. doi: 10.3390/ph16060829 (PMC10303075; doi:10.3390/ph16060829)
Supplement: Supplementary file 1 [file pharmaceuticals-16-00829-s001.zip › pharmaceuticals-2393527-supplementary.pdf]

SUPPLEMENTARY MATERIALS

In Silico Development of Novel Benzofuran-1,3,4-Oxadiazoles Lead Inhibitors of M. tuberculosis Polyketide Synthase13 Enzyme

Ali Irfan[1], Shah Faisal [2], Ameer Fawad Zahoor [1,*] Razia Noreen [3], Sami A. Al-Hussain [4,*], Burak Tuzun [5], Rakshanda Javaid [1], Ahmed A. Elhenawy [6,7], Magdi E. A. Zaki [4], Sajjad Ahmad[8] **and Magda H. Abdellattif [9]**

[1] Department of Chemistry, Government College University Faisalabad, Faisalabad 38000,Pakistan; raialiirfan@gmail.com; fawad.zahoor@gcuf.edu.pk; rakshanda880@gmail.com
[2] Department of Chemistry, Islamia College University Peshawar, Peshawar 25120, Pakistan; faisalvbs@gmail.com
[3] Department of Biochemistry, Government College University Faisalabad, Faisalabad 38000,Pakistan; razianoreen@hotmail.com
[4] Department of Chemistry, College of Science, Imam Mohammad Ibn Saud Islamic University (IMSIU), Riyadh 13623, Saudi Arabia; sahussain@imamu.edu.sa; mezaki@imamu.edu.sa
[5] Plant and animal production department, Technical Sciences Vocational School of Sivas, Sivas Cumhuriyet University, Sivas-58140, Turkey; theburaktuzun@yahoo.com
[6] Chemistry Department, Faculty of Science, Al-Azhar University, Nasr City, Cairo 11884, Egypt
[7] Chemistry Department, Faculty of Science and Art, AlBaha University, Mukhwah, Al Bahah 65731, Saudi Arabia; elhenawy_sci@hotmail.com (AAE.)
[8] Department of Health and Biological Sciences, Abasyn University, Peshawar 25000, Pakistan; sajjad.ahmad@abasyn.edu.pk (S.J)
[9] Department of Chemistry, College of Science, Taif University, PO Box 11099, Taif 21944, Saudi Arabia; M.hasan@tu.edu.sa
* Correspondence: Correspondence: fawad.zahoor@gcuf.edu.pk; (A.F.Z.); Tel.: +923336729186; sahussain@imamu.edu.sa (S.A.Al-H)

Table of contents

Entry	Name of Table	Page No.
S1	The binding affinities of benzofuran-1,3,4-oxadiazoles **BF10-BF16**	2
S2	ADMET profiles of the synthesized benzofuran-1,3,4-oxadiazoles **BF10-16 and TAM-16**	3
S3	Drug-likeness and Medicinal chemistry profiles of benzofuran-1,3,4-oxadiazoles **BF10-16** and **TAM-16**	4

Table S1: The binding affinities of benzofuran-1,3,4-oxadiazoles **BF10-BF16** with the Mtb Pks13 enzyme.

Benzofuran-1,3,4-oxadiazoles	Binding Affinities with Pks13
**BF10**	-11.49 kcal/mol
**BF11**	-10.94 kcal/mol
**BF12**	-11.10 kcal/mol
**BF13**	-10.90 kcal/mol
**BF14**	-12.01 kcal/mol
**BF15**	-11.08 kcal/mol
**BF16**	-12.39 kcal/mol
**TAM-16**	-14.61 kcal/mol

Table S2: ADMET profiles of the synthesized benzofuran-1,3,4-oxadiazoles **BF10-16 and TAM-16**

Compounds	Carcinogenicity	HIA+ Values	Lipophilicity (iLogP)	CYP450 3A4 Inhibitor/Substrate	Log S (ESOL) H_2O Solubility	P-gp Substrate	Renal OCTs
**BF10**	None	1.00	2.73	Substrate	-5.52 Moderately Soluble	No	Non-inhibitor
**BF11**	None	1.00	2.79	Substrate	-3.29 Soluble	No	Non-inhibitor
**BF12**	None	1.00	3.28	Substrate	-5.07 Moderately Soluble	No	Non-inhibitor
**BF13**	None	1.00	3.54	Substrate	-4.54 Moderately Soluble	No	Non-inhibitor
**BF14**	None	1.00	3.68	Substrate	-4.77 Moderately Soluble	No	Non-inhibitor
**BF15**	None	1.00	3.57	Substrate	-5.07 Moderately Soluble	No	Non-inhibitor
**BF16**	None	1.00	3.48	Substrate	-3.75 Soluble	No	Non-inhibitor
**TAM-16 (Standard)**	None	0.993	2.48	Substrate	-3.72 Moderately Soluble	Yes	Non-inhibitor

Table S3. Drug-likeness and Medicinal chemistry profiles of benzofuran-1,3,4-oxadiazoles **BF10-16** and **TAM-16**

Compounds	Bioavailability Score	PAINS Alerts	Accept Lipinski's Rule Yes/No	Pfizer Rule	Golden Triangle Rule	TPSA
**BF10**	0.55	None	Yes	complied	Accepted	106.46 Å^2
**BF11**	0.55	None	Yes	complied	Accepted	118.93 Å^2
**BF12**	0.55	None	Yes	complied	Accepted	106.46 Å^2
**BF13**	0.55	None	Yes	complied	Accepted	115.69 Å^2
**BF14**	0.55	None	Yes	complied	Accepted	115.69 Å^2
**BF15**	0.55	None	Yes	complied	Accepted	106.46 Å^2
**BF16**	0.55	None	Yes	complied	Accepted	97.67 Å^2
**TAM-16**	0.55	1-alert	Yes	complied	Accepted	85.94 Å^2
